# Pediatric and Adult Low-Grade Gliomas: Where Do the Differences Lie?

**DOI:** 10.3390/children8111075

**Published:** 2021-11-22

**Authors:** Ladina Greuter, Raphael Guzman, Jehuda Soleman

**Affiliations:** 1 Department of Neurosurgery, University Hospital of Basel, 4031 Basel, Switzerland; raphael.guzman@usb.ch (R.G.); jehuda.soleman@gmail.com (J.S.); 2 Department of Neurosurgery, King’s College Hospital, NHS Foundation Trust, London SE5 9RS, UK; 3 Division of Pediatric Neurosurgery, University Children’s Hospital of Basel, 4056 Basel, Switzerland; 4 Faculty of Medicine, University of Basel, 4056 Basel, Switzerland

**Keywords:** pediatric low-grade glioma, malignant transformation

## Abstract

Two thirds of pediatric gliomas are classified as low-grade (LGG), while in adults only around 20% of gliomas are low-grade. However, these tumors do not only differ in their incidence but also in their location, behavior and, subsequently, treatment. Pediatric LGG constitute 65% of pilocytic astrocytomas, while in adults the most commonly found histology is diffuse low-grade glioma (WHO II), which mostly occurs in eloquent regions of the brain, while its pediatric counterpart is frequently found in the infratentorial compartment. The different tumor locations require different skillsets from neurosurgeons. In adult LGG, a common practice is awake surgery, which is rarely performed on children. On the other hand, pediatric neurosurgeons are more commonly confronted with infratentorial tumors causing hydrocephalus, which more often require endoscopic or shunt procedures to restore the cerebrospinal fluid flow. In adult and pediatric LGG surgery, gross total excision is the primary treatment strategy. Only tumor recurrences or progression warrant adjuvant therapy with either chemo- or radiotherapy. In pediatric LGG, MEK inhibitors have shown promising initial results in treating recurrent LGG and several ongoing trials are investigating their role and safety. Moreover, predisposition syndromes, such as neurofibromatosis or tuberous sclerosis complex, can increase the risk of developing LGG in children, while in adults, usually no tumor growth in these syndromes is observed. In this review, we discuss and compare the differences between pediatric and adult LGG, emphasizing that pediatric LGG should not be approached and managed in the same way as adult LCG.

## 1. Introduction

Brain tumors are the most common solid cancer in children, with an incidence of 5.7 per 100,000 [1]. Gliomas comprise approximately a third of these tumors, with two thirds of gliomas classified as low-grade gliomas (pLGG) in children and adolescents [2]. Pediatric LGGs are defined as astrocytoma World Health Organization (WHO) grade I and II, and include several different pathological subtypes, such pilomyxoid astrocytomas, oligodendrogliomas, gangliogliomas or supendemal giant cell astrocytomas [3,4,5]. In adults, low-grade gliomas (aLGG) are less frequent, making up only 15–25% of all gliomas [6]. In children, pilocytic astrocytomas (PAs), classified as WHO grade I tumors, are the most common pLGG (65%), while in adults, WHO grade II gliomas are more common [6,7,8]. In aLGG, the current EFNS-EANO guidelines recognize WHO grade II gliomas as diffuse infiltrative gliomas and they feature a different risk profile compared to PA [7,9]. In children, most pLGGs occur in the cerebellum, while in adults, they mostly occur in the supratentorial compartment [4]. The ten-year overall survival (OS) rate is over 90% for pLGG, while in aLGG, median survival is approximately 7 years with a ten-year OS lower than 60% [10,11,12,13]. In adults, many LGGs undergo malignant transformation and become high-grade gliomas (aHGG), while this transformation is rarely observed in pLGG [9,13,14,15,16,17]. The different biology of tumors in pediatric and adult cohorts dictates different treatment strategies and prognostic outlooks [8].

In this review, we discuss and compare the differences between pLGG and aLGG, emphasizing that LGG in children and adults are two different tumor entities and that the same tumor should be approached individually in the different age groups. The purpose of this review is to offer a brief but comprehensive overview of the differences and similarities between LGG in children and adults regarding its histology, molecular biology, and treatment, as well as outcome. This review is mainly addressed to fellow neurosurgeons, neuro-oncologists, and pediatricians, or any medical professionals treating patients with these conditions.

## 2. Histology and Anatomical Location

**Figure 1:**
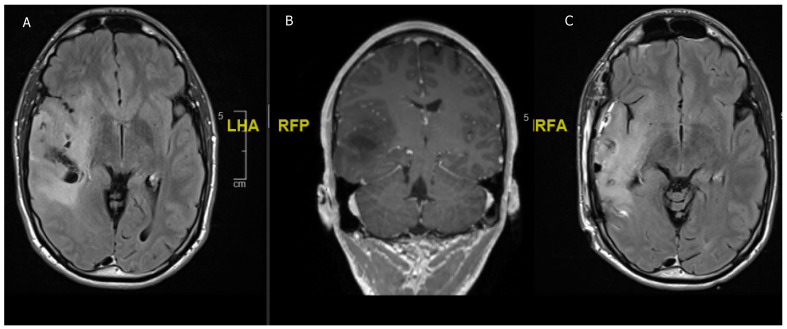
A 21-year-old male, presenting with a generalized seizure. (**A**) Flair hyperintense fronto-temporal tumor of 8.6 × 4.5 × 5 cm with central hypointensity suggestive of aLGG. (**B**) T1 hypointense tumor surrounded by the vessels of the sylvian fissure. (**C**) Postoperative imaging after awake fronto-temporal craniotomy and partial tumor resection due to language impairment intraoperatively.

In children, most LGGs are pilocytic astrocytoma (65%), followed by LGG not otherwise specified (NOS) in 21% of cases. Approximately, three quarters of all LGGs in children are WHO grade I [8]. In adults, it is the opposite, with over three quarters of aLGG being WHO grade II [6]. PLGGs occur in 60%, either in the supra- or in the infratentorial compartment, while aLGGs occur in 60% of cases supratentorially, with 80% in eloquent regions [6,18]. In children, certain tumor locations, such as the tectum or the dorsal brainstem, are pathognomonic for pLGG, specifically pilocytic astrocytoma (PA), and it is disputed whether these tumors even require a biopsy to confirm the diagnosis, since surgery around the brainstem has a rate of postoperative morbidity of up to 30% [9,19]. Therefore, if hydrocephalus is apparent, endoscopic biopsy is obtained simultaneously to an endoscopic third ventriculostomy (ETV), which is indicated in order to treat the aqueduct stenosis caused by the tumor [20,21,22]. A classic location for aLGG is the insular region, which presents specific challenges (Figure 1). These tumors are very closely located to the middle cerebral artery and are surrounded by its branching vessels in a highly eloquent region, which often involves speech, motor, and limbic functions [23]. Approximately 6% of all pLGGs show dissemination either in other parts of the brain or the spine, while in adults dissemination is only observed in high grade gliomas [24,25]. However, the benefit of a whole neuroaxis scan at diagnosis and follow-up is still controversially discussed in pLGG [26].

## 3. Predisposition Syndromes Associated with LGG

Certain hereditary syndromes, such as Neurofibromatosis type I (NF-1) and Tuberous Sclerosis complex (TSC), are associated with LGG. Since these tumors tend to grow and become symptomatic mainly in the pediatric age, the dilemma of how to diagnose and when to treat these patients mostly affects pediatric oncologists, pediatric neurologists, and pediatric neurosurgeons; to a lesser extent, this dilemma also affects clinicians dealing with adult NF-1 or TSC patients [27].

### 3.1. Neurofibromatosis Type 1

Patients with NF-1 are at risk of developing optic pathway glioma, which usually involves tumor growth during childhood but not during adult life [27]. Around 6% of all NF-1 patients develop optic pathway glioma (OPGs), with a peak of incidence at the age of 3–4 years [28,29]. These OPGs show a more benign course than their sporadic counterparts [28,29,30]. The primary treatment goal for these children is visual preservation [27]. Only around 1% of all NF-1 patients additionally develop brainstem gliomas with a risk of hydrocephalus due to secondary obstructive hydrocephalus [22,29].

### 3.2. Tuberous Sclerosis Complex

**Figure 2:**
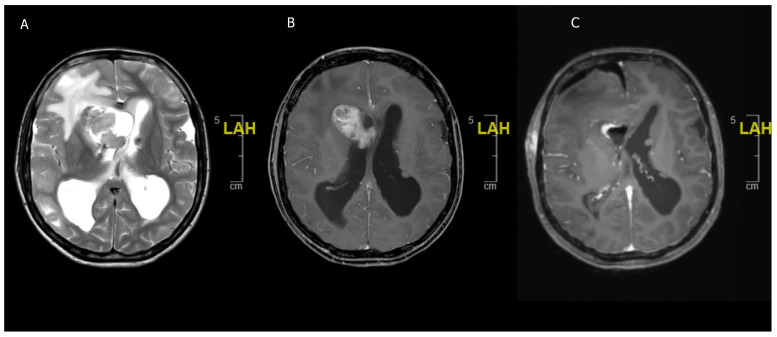
A 16-year-old girl previously diagnosed with TSC and presenting with acute headaches and vomiting. (**A**) Acute obstructive hydrocephalus and edema due to T2 hypointense lesion in the right frontal horn. (**B**) Contrast enhancing lesion in the right frontal horn suggestive of SEGA in the context of TSC. (**C**) Postoperative scan with reduction of ventricular size and showing complete resection of the SEGA.

The TSC negatively regulates the mTOR pathway, resulting in typical subependymal giant cell astrocytoma (SEGA) in 20% of all patients, and only a small number of reports of SEGA without the clinical features of TSC exist (Figure 2) [31,32]. These tumors mostly occur in children and young adults and are unlikely to occur in adulthood [30]. If they increase in size, they can cause seizures or obstructive hydrocephalus, which is when surgical excision is recommended, although some reports showed that mTOR inhibitors are sufficient for hydrocephalus treatment as well as seizure reduction and control [9]. Moreover, preoperative treatment with mTOR inhibitors can reduce the vascularity of SEGAs, which is beneficial for surgical resection [33,34]. In most cases, the tumor can be medically treated with mTOR inhibitors, which can prevent disease progression in children >3 years of age and has also been shown to be effective terms of cognition, development, and seizure control in TSC children [34,35,36].

For both NF-1 associated OPGs and TSC-associated SEGAs, a diagnosis can be made based on typical MRI features, and a biopsy is not obligatory to confirm histopathological diagnosis before starting medical therapy [35]. This stands in contrast to the usual treatment paradigm, which requires the performance of a biopsy before starting therapy for any other presumed LGG, especially in adults. However, in the context of a predisposition syndrome, omitting a biopsy before initi ating treatment is accepted by most pediatric neurooncologist, neurologists, and neurosurgeons [35] (Table 1).

NF-1 is associated with OPG, which follows a more benign course than its sporadic counterpart. The primary treatment goal is visual preservation. Around every fifth patient with TSC develops a SEGA, which is primarily treated with mTOR inhibitors. In both, diagnosis is often made based on imaging features only.

## 4. Treatment of pLGG vs. aLGG

### 4.1. Surgery

In both children and adults, the mainstay of therapy is surgical resection. Since most patients present with symptoms due to mass effect over the course of several months, the primary goal of therapy is volume reduction [5]. Surgery is the most effective treatment to achieve a volume reduction of the tumor, whilst a histopathological diagnosis can also be reached. In fact, for pLGG, a gross total resection (GTR) of the tumor often means the patient is cured of the disease (since most pLGGs are WHO grade I), while for aLGG (which are mostly WHO grade II), unfortunately, even after GTR, the chances of recurrence and/or malignant transformation remain relatively high [37]. As mentioned above, pLGG and aLGG typically occur at different anatomical locations, which is why the surgical approach and skillset of surgeons differs depending on whether they treat children or adults [6]. Due to the highly eloquent region of the insula, most neurosurgeons treating aLGG are trained in performing awake craniotomies, which are applied in around a third of all insular aLGGs but are rarely applied in children [23,38]. On the other hand, pediatric neurosurgeons often encounter posterior fossa pLGG or exophytic brainstem pLGG and require a specific skillset for these approaches, as well as treating obstructive hydrocephalus or tumor biopsies with endoscopic procedures (e.g., ETV) [39]. Intraventricular tumors (e.g., pLGG within the third ventricle, thalamic pLGG) requiring an endoscopic or transventricular approach, are also more often encountered in children than in adults^40^ [40]. It is therefore clear that LGGs show anatomical differences in different age groups which directly influences and dictates their treatment strategy. Typically, in children, posterior fossa syndrome, including cerebellar mutism occurs in approximately a third of patients undergoing infratentorial tumor resection, while this is an extremely rare complication after posterior fossa surgery in adults [41]. Some published reports suggest that children recover faster and better after epilepsy surgery compared to adults, while no clear evidence on this matter exists in tumor surgery [42,43,44,45]. In awake tumor surgery, differences in neurostimulation are observed between children and adults, which is most probably due to the different myelination in the cortical and subcortical tracts between the two age groups, indicating different levels of plasticity/brain development [46].

Several studies have shown that gross total resection (GTR) correlates with increased progression-free survival (PFS) and overall survival (OS) in both children and adults [4,47,48,49]. Near-total resection with minimal residual (<1.5 cm^3^) was suggested to have a similar outcome to complete resection in both age groups, but the results are controversially discussed in children [4,48]. In a case series by Wisoff et al., eight-year PFS was 93% ± 1.5% if GTR was achieved, 56% ± 5.2% with a residual <1.5 cm^3^ and 45% ± 6.2% with a tumor residual >1.5 cm^3^ [4]. Second-look surgery is highly recommended for children with a postoperative residual or recurrent tumor amenable to resection [35]. A study by Saunders et al. investigating pLGG in the posterior fossa showed that only 13% of all children with a GTR presented with a recurrent tumor, resulting in a six times lower risk of tumor progression than after a subtotal resection [50]. Similar results were also demonstrated for supratentorial pLGG [4]. Of all recurrent pLGGs, two thirds required subsequent therapies—either surgery or radio- and chemotherapy [50]—underlining the importance of regular radiographic follow-ups. Tumor location is a prime determinant of prognosis, with a significantly worse prognosis for brainstem or optic pathway pLGG, with the exception of OPG in NF-1 patients [4,28−30,49,50]. Unfavorable prognostic factors with regards to overall survival (OS) include subtotal resection, young age, and unfavorable tumor location (e.g., brainstem, or optic pathway) [51,52,53]. In adults, GTR is more likely to be achieved in incidental lesions than in symptomatic lesions, which is most probably due to their non-eloquent location. In a large case series by Gogos et al., GTR was achieved in 57% of incidental and 24% of symptomatic aLGGs, with a significantly shorter survival of symptomatic aLGGs (median 14.6 years) [54]. For adults, early resection resulted in a longer OS than observation and a delayed resection of aLGG [54]. To achieve a complete resection with the total removal of FLAIR abnormalities in aLGG is difficult, as the tumor is non-enhancing and often presents as normal tissue during surgery [7]. If a tumor recurrence or progression is amenable to surgery, an additional resection should be considered [7]. For both age groups, the use of neuronavigation, ultrasound, and neuromonitoring as adjuncts to improve the quality of the resection are highly encouraged [7,35]. The use of 5-aminolevulinic acid (5-ALA), a commonly used tool to optimize the extent of resection in high grade glioma, is often less reliable in aLGG. Fluorescence induced by 5-ALA induced is observed in a variable amount in different case series of aLGG, with an average of 24.6% positivity limiting its use [55]. Similarly to pHGG, in pLGG, 5-ALA is not used routinely and therefore no data exist on its induced florescence [56].

Gross total resection is the primary therapy for both pediatric and adult LGG and significantly improves OS. However, in pLGG, GTR seems to lead to better prognosis than in adults. This most probably due to differences in histology and tumor biology.

### 4.2. Adjuvant Therapy

Children with recurrent tumors, radiographic growth of remaining tumors, or tumors in unfavorable locations inaccessible to future surgery, are advised to undergo adjuvant treatment [35]. Chemotherapy is the first-line adjuvant therapy and preferred over radiotherapy (RT) [35,57,58]. Few trials regarding different chemotherapy regiments exist [35,51,52,57,59,60] (Table 2). Children with neurofibromatosis type 1 (NF-1) and associated pLGG showed a much higher response to chemotherapy (carboplatin, vincristine) with a PFS of 69% compared to 39% for the remaining pLGG [61]. In the case of NF-1 and non-resectable tumors, chemotherapy without a prior biopsy is recommended as first-line therapy [35].

The current practice in adults is similar. Adjuvant therapy is not considered for low-risk aLGG, with low-risk defined as age <40 years and a total resection of the FLAIR abnormalities in the postoperative MRI. For radiological tumor progression (>25% radiological increase of tumor size) or recurrence not amenable to surgery, adjuvant therapy is usually suggested [54,67,68,69]. However, in adults, the recommended type of chemotherapy differs from pediatric patients, and for adults, concomitant radiotherapy (RT) is often suggested [7]. Several chemotherapy regimens have been described for aLGG and are summarized in Table 2 [7,65,66].

Due to the side effects of radiotherapy, especially in younger children, including neurocognitive impairment, developmental delay, hearing dysfunction, vasculopathy, malignant transformation, or secondary tumors [35,57,58], RT only plays a role in recurrences after primary therapy or as salvage therapy in pLGG [35,57,58]. RT can be administered at any time after chemotherapy and is not impacted by the regimen administered beforehand [35]. The recommended radiotherapy dose for pLGG is 45–50.4 Gray (Gy) in 1.8 Gy fractions, although this ultimately depends on the tumor and its location. The OS of subtotal resected tumors in unfavorable locations showed a similar survival time when treated with postoperative radiotherapy (RT) compared to pLGG treated with GTR and surveillance only [49]. Craniospinal irradiation (CSI) is only administered for disseminated disease and is rarely used for pLGG [35]. Proton beam therapy (PBT) offers an advantage over conventional RT in eloquent tumor locations, such as optic or hypothalamic-pituitary pathways, since it reduces the dose to the surrounding normal brain by 1.5–3 times [65]. A study by Greenberger et al. showed that PBT demonstrated similar tumor control rates compared to conventional RT but showed fewer endocrine and neurocognitive side effects [70]. However, the superiority of PBT compared to conventional RT is still controversially discussed in research and current guidelines [35,71]. Patients with NF-1 were shown to suffer more frequently and severely from neurocognitive decline after RT; hence, it should only be applied cautiously in this cohort [35,58]. In aLGG, two randomized trials were conducted for RT. One compared watch-and-wait to RT after surgery, while the other compared early versus late (after progression) RT; in both trials, no difference in OS was detected [72,73]. Hence, RT is not administered for low-risk aLGG, but at progression, it is more commonly used as an adjuvant therapy concomitant with chemotherapy.

## 5. Molecular Genomics and Targeted Therapies

In recent years, advances in molecular tumor research identified abnormalities in the mitogen-activated protein kinase/extracellular-signal- regulated kinase (MAPK/ERK) activation pathway, due to a mutation or fusion of the BRAF gene, that were present in pLGG but not in aLGG [74,75,76]. A similar change of the MAPK pathway is observed in NF-1, predisposing patients to pLGGs [62]. Up to 84% of pLGGs harbor a driver mutation, of which KIAA1549-BRAF (35%) mutation was the most commonly found, followed by the BRAFV600E and NF-1 mutations (17%) [76,77]. Rare alterations additionally occur in up to 17% of cases [76]. The different driver mutations influence the outcome and can offer therapeutic targets for novel therapies [74,78,79,80]. KIAA1549-BRAF-mutated pLGG are mostly found in the cerebellum and present with a higher 5-year progression-free rate as well as a higher 10-year overall survival rate compared to BRAFV600E-mutated pLGGs (69% vs. 52% and 97% vs. 89%) [76]. By contrast, BRAFV600E rarely occurs in pediatric high-grade gliomas, where it predicts a better outcome [63,81].

**Figure 3:**
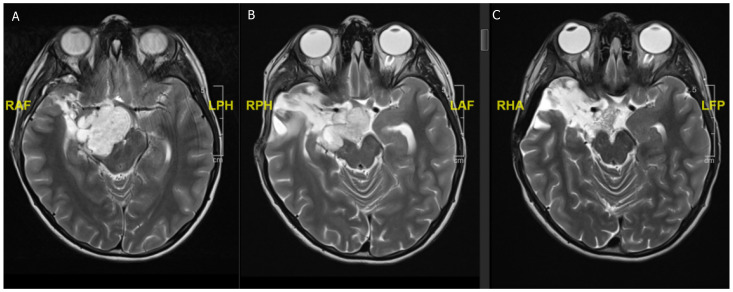
An 11-year-old boy presenting with headaches and blurry vision. (**A**) Preoperative MRI scan showing a 6.1 × 4.0 × 3.4 cm T2 hyperintense and contrast enhancing (not shown) lesion suggestive of (non-NF) optic pathway glioma. (**B**) Postoperative MRI scan after partial resection with tumor rest in the optic pathway. Histology confirmed a BRAF600 mutated pilocytic astrocytoma. (**C**) Follow-up MRI scan after 4 weeks of MEK inhibitor treatment showing a nearly complete regression of the tumor.

Most pLGGs, even if they do not include one of the typical driver mutations, contain mutations affecting the MAP-Kinase pathway. This could make them possible targets for BRAF/MEK inhibitor therapy [64,76]. The two best-known BRAF/MEK inhibitors currently being investigated in pLGG are selumetinib and trametinib, with skin toxicity being the most frequently described adverse event of the treatment [74,78,82,83] (Figure 3).

PLGGs harboring KIAA1549-BRAF mutations are more responsive to BRAF inhibitors than BRAFV600E-mutated tumors [77]. A recent phase II trial for recurrent or progressive pLGG showed that patients treated with selumetinib had a two-year PFS of 78 ± 8.5% and that visual fields and acuity improved or remained stable in the majority of patients, concluding that selumetinib leads to prolonged disease stability [78]. A current prospective trial investigating trametinib in pLGG is ongoing; the results are yet to be published (NCT02124772). In two retrospective cohort studies with a total of 28 patients, 12 patients showed a minor or partial response, while 16 patients achieved stable disease when treated with trametinib [82,83]. In one of the studies, around a third showed disease progression over the course of treatment, while in the other cohort, all the patients achieved disease control [82,83]. There is currently a trial ongoing comparing MEK/BRAF inhibitors to a CV regimen in pLGG, focusing on overall response rate (ORR), PFS, and OS; the results are yet to be published (NCT02684058). Some studies have observed a paradoxical activation of tumor growth with BRAF/MEK inhibitor treatment, especially in KIAA1549-BRAF- and NF-1-mutated pLGG [64,84]. This might prompt even more specific therapies for the distinct mutations [84]. Another challenge after the successful initiation of BRAF/MEK inhibitors is to decide when to discontinue treatment, weighing the risk for tumor recurrence and treatment side effects against each other. Tumor progression is often observed after the termination of BRAF/MEK inhibitors [82,83]. Target therapy trials for LGG are currently only carried out within the pediatric population.

In children and adults, adjuvant therapy is administered if radiographic tumor progression (>25% of tumor volume) or recurrences not amenable to surgery are apparent. In pLGG, the adjuvant therapy of choice is a regimen of CV, radiotherapy, or BRAF/MEK inhibitors used as salvage therapy. On the other hand, in aLGG, a common practice is the use of concomitant RT with temozolomide, despite disputed evidence for TMZ compared to PCV. Due to the molecular biology of pLGG, MEK inhibitors are a novel treatment method, with promising results and ongoing trials.

### 5.1. Malignant Transformation

Malignant transformation (MT) of pLGG is a rare phenomenon, which occurs in 2.9–6.7% of all patients based on a small number of case series [15,17,85,86,87]. It is assumed that the histopathology and molecular biology (MAPK/ERK deregulation, PI3K/AKT aberrations) might influence the risk of MT, but no clear pattern that can be translated into clinical practice has yet been identified [15,16,17]. It has been suggested that MT in children is associated with the prior administration of chemotherapy or RT [5,7,87].

In adults, MT occurs much more frequently, with rates ranging between 13–86% of all aLGGs. Similar to children, MT seems to be associated with the administration of adjuvant therapy [37,88,89,90]. Furthermore, several reports describe a rapid growth and higher rate of malignant transformation of aLGG in pregnant women [91,92,93]. This is probably due to hemodynamic, as well as molecular changes. One study showed that progesterone can accelerate tumor growth, while another study found a correlation between elevated insulin-like growth factor-1 (IGF-1) levels, which are elevated during pregnancy, and the growth of astrocytoma [91,94,95]. No reports about accelerated MT in puberty could be found, and previous reports about treatment with growth hormone accelerating tumor growth are controversially discussed [15,96]. In adults, IDH and p53 mutations might promote MT, which are different molecular mechanisms to those present in pLGG [8,75,97]. It is highly debatable whether teenagers and young adults should be considered as children (counting as a low-risk group) or as adults. Common practice indicates that if the tumor resembles a typical childhood tumor, the management should be guided accordingly, or the other way round for tumors resembling aLGG [9].

This differences between the age groups underline the molecular differences between these two groups [5,88,89,98]. Due to the higher rate of MT in adults, early surgery aiming for gross total resection is also warranted in incidental tumors, while in children the watch-and-wait strategy still seems to be justified for small incidental findings [9,15]. In the case of easily accessible lesions in children, surgical excision is feasible and can be discussed [9].

Malignant transformation is a rare event in pLGG and might be associated with adjuvant therapy, while it occurs in most aLGG, most probably due to the different tumor biology.

### 5.2. Spontaneous Regression in pLGG

**Figure 4:**
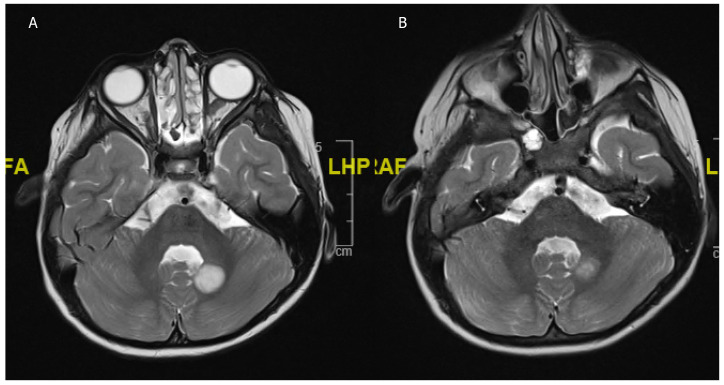
An 8 year-old girl undergoing treatment for congenital scoliosis. (**A**) MRI head carried out in the setting of her scoliosis work-up showed an incidental finding of a T2 hyperintense cerebellar lesion (**B**) Interval scan after 6 months showed a spontaneous regression of the lesion. No resection or biopsy was attempted.

The phenomenon of spontaneous regression has been described in some reports on pLGG but it is basically non-existent in adults. In a case series by Ogiwara, a total of 30% of all partially resected cerebellar pLGG demonstrated spontaneous regression over a median time of 11.9 months [99]. Similar rates (32.5–48%) of spontaneous regression or arrested growth have been described in other case series of cerebellar pLGG [100,101] (Figure 4). This could warrant the decision not to chase the tumor into eloquent regions, such as the peduncle or parts of the brainstem, to avoid postoperative morbidity [99]. Telomerase shortening and the induction of apoptosis are hypothesized to cause growth arrest and the telomere length could offer some prognostic value in pLGG; however, further studies are needed to elucidate this matter [102].

Spontaneous regression is observed in around one third of pLGGs but not in aLGG. This warrants the concept of avoiding radical tumor resection in eloquent regions in children with suspected or confirmed LGG.

## 6. Conclusions

Pediatric and adult low-grade gliomas differ in their pathology as well as their anatomical location, dictating the terms of their management. Predisposition syndromes, such as NF-1 or TSC, are associated with specific types of low-grade glioma, which occur during childhood. In these cases, medical therapy is often initiated without obtaining histopathological samples.

Surgical resection with the aim of gross total resection is the primary treatment in pediatric and adult low-grade gliomas. In both age groups, gross total resection is associated with longer overall survival, while in the pediatric population it is higher than in the adult population. Adjuvant therapy is only advocated for progressive or recurrent low-grade glioma in both age groups. In children, chemotherapy with carboplatin and vincristine is the standard adjuvant therapy, while in adults concomitant temozolomide and radiotherapy are administered. Novel therapies, such as MEK inhibitors, were shown to be effective in progressive pediatric low-grade gliomas and are currently being investigated in comparison to chemotherapy. Currently, these agents do not play a role in adult low-grade glioma due to the different molecular biology of these tumors. In pediatric low-grade glioma, malignant transformation is a rare event and mostly associated with previous chemo- and/or radiotherapy, while most adult low-grade gliomas undergo malignant transformation. This again underlines the differences between low-grade gliomas in children and adults regarding their biology and behavior. Due to the various differences in diagnosis and treatment, children with low-grade gliomas should only be treated in centers with pediatric oncologists, pediatric neurologists, and pediatric neurosurgeons.

## Figures and Tables

**Table 1:** Characteristics of pediatric and adult LGG.

	pLGG	aLGG
**Anatomical Location**	Supra- and infratentorial each 30%	Supratentorial (eloquent regions) 80%
**Most common histopathology**	WHO I: 74%	WHO I: 10–15%
	WHO II: 26%	WHO II: 85–90%
	Pilocytic astrocytoma 65%	Diffuse LGG 60%
**Primary treatment**	Surgery,	Surgery,
	GTR increases OS	GTR increases OS
**Indication for adjuvant therapy**	Radiographic progression (>25% of volume) or recurrence not amenable to re-resection	Radiographic progression or recurrence not amenable to re-resection
**Chemotherapy**	carboplatin and vincristine (CV)	Temozolomide (TMZ) often preferred to procarbazine, lomustine, and vincristine (PCV)
**Radiotherapy**	Salvage therapy, 45–50.4 Gy in 1.8 Gy fractionsConsider proton beam therapy if feasible	Concomitant with chemotherapy with 50.4–54 Gy in 1.8Gy fractions
**Novel therapies**	BRAF/MEK inhibitors (trametinib, selumetinib), ongoing trials	-
**10-year OS (%)**	>90%	~60%
**Molecular alterations**	BRAF600 17%	IDH mutant 70%
**Malignant transformation**	Extremely rare (2.9–6.7%), after Chemo or RT might be higher	Common, up to 86%
**Associated syndromes**	NF-1, TSC NF-1 associated OPG highly sensitive to chemotherapy but more side effects with RT TSC associated SEGA mTOR therapy	-
**Prognostic factors**	Location (optic pathway, brainstem worse prognosis, OPG with NF-1 very good prognosis), GTR (better prognosis), young age (worse prognosis)	Location (eloquent worse prognosis), GTR (better prognosis), diffuse LGG WHO II (worse prognosis), age <40 years (better prognosis)

Abbreviations: OS = overall survival, NF-1 = neurofibromatosis, TSC = tuberous sclerosis complex, GTR = gross total resection, MEK inhibitor = mitogen-activated protein kinase, IDH = isocitrate dehydrogenase, RT = radiotherapy, LGG = low grade glioma, OPG = optic pathway glioma.

**Table 2:** Different randomized controlled trials comparing adjuvant regiments in LGG and ongoing trials for MEK inhibitors.

Study	Agents	PFS	OS	Comments
**Pediatric LGG**				
Ater et al., 2012 [51]	carboplatin and vincristine (CV) **vs.** thioguanine, procarbazine, lomustine, and vincristine (TPCV)	5 year PFS 39% ± 4% **vs.** 52% ± 5%	5 year OS 86% ± 3% **vs.** 87% ± 7%	
Gnekow et al., 2017 [52]	carboplatin & vincristine (CV) **vs.** carboplatin, vincristine & etoposide (CVE)	5-year PFS 46% ± 3.5% **vs.** 45% ± 3.5%	5 year OS 86% ± 2.1% in both groups	
**BRAF/MEK Inhibitors**				
Fangusaro J et al., 2019 [62]	Selumetinib in presence of recurrent, refractory or progressive pLGG	36–40% reach partial response	Not applicable	
NCT02124772 (ongoing)	Trametinib pharmacokinetics study	Results expected 2021	Results expected 2021	
NCT04485559 (ongoing)	Trametinib and everolimus dosage study	Results expected 2023	Results expected 2023	
Selt et al., 2020 [63] (retrospective)	Trametinib in progressive pLGG	18 patients, 6 partial responses, 2 minor responses, 10 stable diseases (SD), 100% disease control under therapy	-	
Manoharan et al., 2020 [64] (retrospective)	Trametinib in progressive pLGG	10 patients, 2 partial response, 2 minor response, 6 stable disease	-	
**Adult LGG**				
Baumert et al., 2016 [65]	Temozolomide (TMZ) **vs.** Radiotherapy	4-year PFS 39 months (IQR:16–46) **vs.** 46 months (IQR:19–48)	-	
Buckner et al., 2016 [66]	Radiotherapy **vs.** Radiotherapy and procarbazine, lomustine, vincristine	10-year PFS 21% **vs.** 51%	Overall survival 7.8 years **vs.** 13.3 years	Due to side effects, PCV is often replaced with TMZ by clinicians, despite disputed evidence
NCT00887146 (ongoing)	Radiotherapy & TMZ **vs.** Radiotherapy and procarbazine, lomustine, vincristine	Results expected 2025	Results expected 2025	

## Data Availability

Not applicable.

## References

[1] Ostrom Q.T., Gittleman H., Truitt G., Boscia A., Kruchko C., Barnholtz-Sloan J. (2018). CBTRUS statistical report: Primary brain and other central nervous system tumors diagnosed in the United States in 2011–2015. Neuro-Oncology.

[2] Diwanji T.P., Engelman A., Snider J.W., Mohindra P. (2017). Epidemiology, diagnosis, and optimal management of glioma in adolescents and young adults. Adolesc. Heal. Med. Ther..

[3] Louis D.N., Perry A., Reifenberger G., von Deimling A., Figarella-Branger D., Cavenee W.K., Ohgaki H., Wiestler O.D., Kleihues P., Ellison D.W. (2016). The 2016 World Health Organization classification of tumors of the central nervous system: A summary. Acta Neuropathol..

[4] Wisoff J., Sanford R.A., Heier L.A., Sposto R., Burger P.C., Yates A.J., Holmes E.J., Kun L.E. (2011). Primary neurosurgery for pediatric low-grade gliomas: A prospective multi-institutional study from the children’s oncology group. Neurosurgery.

[5] Collins K.L., Pollack I.F. (2020). Pediatric Low-Grade Gliomas. Cancers.

[6] Rasmussen B.K., Hansen S., Laursen R.J., Kosteljanetz M., Schultz H., Nørgård B.M., Guldberg R., Gradel K. (2017). Epidemiology of glioma: Clinical characteristics, symptoms, and predictors of glioma patients grade I–IV in the the Danish Neuro-Oncology Registry. J. Neurooncol..

[7] Soffietti R., Baumert B., Bello L., Von Deimling A., Duffau H., Frénay M., Grisold W., Grant R., Graus F., Hoang-Xuan K. (2010). Guidelines on management of low-grade gliomas: Report of an EFNS-EANO* task force: Low-grade gliomas. Eur. J. Neurol..

[8] Bandopadhayay P., Bergthold G., London W.B., Goumnerova L.C., La Madrid A.M., Marcus K.J., Guo D., Ullrich N.J., Robison N.J., Chi S.N. (2014). Long-term outcome of 4040 children diagnosed with pediatric low-grade gliomas: An analysis of the Surveillance Epidemiology and End Results (SEER) database. Pediatr. Blood Cancer.

[9] Soleman J., Kozyrev D., Ben-Sira L., Constantini S., Roth J. (2020). Management of incidental brain tumors in children: A systematic review. Child’s Nerv. Syst..

[10] Claus E.B., Walsh K., Wiencke J.K., Molinaro A.M., Wiemels J.L., Schildkraut J.M., Bondy M.L., Berger M.S., Jenkins R.B., Wrensch M. (2015). Survival and low-grade glioma: The emergence of genetic information. Neurosurg. Focus.

[11] Pignatti F., Bent M.V.D., Curran D., Debruyne C., Sylvester R., Therasse P., Áfra D., Cornu P., Bolla M., Vecht C. (2002). Prognostic factors for survival in adult patients with cerebral low-grade glioma. J. Clin. Oncol..

[12] Wijnenga M.M.J., Mattni T., French P., Rutten G.-J., Leenstra S., Kloet F., Taphoorn M.J.B., Bent M.J.V.D., Dirven C.M.F., Van Veelen M.-L. (2017). Does early resection of presumed low-grade glioma improve survival? A clinical perspective. J. Neurooncol..

[13] Ramakrishna R., Hebb A., Barber J., Rostomily R., Silbergeld D. (2015). Outcomes in reoperated low-grade gliomas. Neurosurgery.

[14] Jakola A.S., Bouget D., Reinertsen I., Skjulsvik A.J., Sagberg L.M., Bø H.K., Gulati S., Sjåvik K., Solheim O. (2020). Spatial distribution of malignant transformation in patients with low-grade glioma. J. Neuro-Oncol..

[15] Soleman J., Roth J., Ram Z., Yalon M., Constantini S. (2017). Malignant transformation of a conservatively managed incidental childhood cerebral mass lesion: Controversy regarding management paradigm. Child’s Nerv. Syst..

[16] Mistry M., Zhukova N., Merico D., Rakopoulos P., Krishnatry R., Shago M., Stavropoulos J., Alon N., Pole J., Ray P.N. (2015). *BRAF* mutation and *CDKN2A* deletion define a clinically distinct subgroup of childhood secondary high-grade glioma. J. Clin. Oncol..

[17] Broniscer A., Baker S.J., West A.N., Fraser M.M., Proko E., Kocak M., Dalton J., Zambetti G.P., Ellison D.W., Kun L.E. (2007). Clinical and molecular characteristics of malignant transformation of low-grade glioma in children. J. Clin. Oncol..

[18] Duffau H., Capelle L. (2004). Preferential brain locations of low-grade gliomas: Comparison with glioblastomas and review of hypothesis. Cancer.

[19] Faulkner H., Arnaout O., Hoshide R., Young I.M., Yeung J.T., Sughrue M.E., Teo C. (2021). The surgical resection of brainstem glioma: Outcomes and prognostic factors. World Neurosurg..

[20] Li K.W., Roonprapunt C., Lawson H.C., Abbott I.R., Wisoff J., Epstein F., Jallo G.I. (2005). Endoscopic third ventriculostomy for hydrocephalus associated with tectal gliomas. Neurosurg. Focus.

[21] Kobayashi N., Ogiwara H. (2016). Endoscopic third ventriculostomy for hydrocephalus in brainstem glioma: A case series. Child’s Nerv. Syst..

[22] Roth J., Ber R., Constantini S. (2019). Neurofibromatosis type 1-related hydrocephalus: Treatment options and considerations. World Neurosurg..

[23] Di Carlo D.T., Cagnazzo F., Anania Y., Duffau H., Benedetto N., Morganti R., Perrini P. (2020). Post-operative morbidity ensuing surgery for insular gliomas: A systematic review and meta-analysis. Neurosurg. Rev..

[24] Chamdine O., Broniscer A., Wu S., Gajjar A., Qaddoumi I. (2016). Metastatic low-grade gliomas in children: 20 years’ experience at St. Jude Children’s Research Hospital: Long-term follow-up of metastatic low-grade gliomas in children. Pediatr. Blood Cancer.

[25] Munshey A., Moore J., MacLean C., Longano A., Goldschlager T. (2017). Cranial pilocytic astrocytoma with spinal drop metastasis in an adult: Case report and literature review. World Neurosurg..

[26] Roth J., Fischer N., Limbrick D.D., Crevecoeur T., Ben-Sira L., Constantini S. (2020). The role of screening spinal MRI in children with solitary posterior fossa low-grade glial tumors. J. Neurosurg. Pediatrics.

[27] Shofty B., Ben Sira L., Constantini S. (2020). Neurofibromatosis 1–associated optic pathway gliomas. Child’s Nerv. Syst..

[28] Campen C.J., Gutmann D.H. (2018). Optic pathway gliomas in neurofibromatosis type 1. J. Child Neurol..

[29] Evans D.G.R., Salvador H., Chang V.Y., Erez A., Voss S.D., Schneider K.W., Scott H.S., Plon S.E., Tabori U. (2017). Cancer and central nervous system tumor surveillance in pediatric neurofibromatosis 1. Clin. Cancer Res..

[30] Malbari F., Lindsay H. (2019). Genetics of common pediatric brain tumors. Pediatr. Neurol..

[31] Adriaensen M.E.A.P.M., Schaefer-Prokop C.M., Stijnen T., Duyndam D.A.C., Zonnenberg B.A., Prokop M. (2009). Prevalence of subependymal giant cell tumors in patients with tuberous sclerosis and a review of the literature. Eur. J. Neurol..

[32] Corlette L., Reid A., Roberts-Thomson S., Christie M., Gaillard F. (2020). Solitary subependymal giant cell astrocytoma: Case report and review of the literature. J. Clin. Neurosci..

[33] Jiang T., Du J., Raynald L., Wang J., Li C. (2017). Presurgical administration of mTOR inhibitors in patients with large subependymal giant cell astrocytoma associated with tuberous sclerosis complex. World Neurosurg..

[34] Northrup H., Aronow M.E., Bebin E.M., Bissler J., Darling T.N., de Vries P.J., Frost M.D., Fuchs Z., Gosnell E.S., Gupta N. (2021). Updated international tuberous sclerosis complex diagnostic criteria and surveillance and management recommendations. Pediatr. Neurol..

[35] Gnekow A.K., Kandels D., Van Tilburg C., Azizi A., Opocher E., Stokland T., Driever P.H., Van Meeteren A.Y.N.S., Thomale U.W., Schuhmann M.U. (2019). SIOP-E-BTG and GPOH guidelines for diagnosis and treatment of children and adolescents with low grade glioma. Klin. Pädiatrie.

[36] Jóźwiak S., Nabbout R., Curatolo P. (2013). Management of Subependymal Giant Cell Astrocytoma (SEGA) associated with Tuberous Sclerosis Complex (TSC): Clinical recommendations. Eur. J. Paediatr. Neurol..

[37] Hervey-Jumper S.L., Berger M.S. (2016). Maximizing safe resection of low- and high-grade glioma. J. Neuro-Oncol..

[38] Sanai N., Polley M.-Y., Berger M.S. (2010). Insular glioma resection: Assessment of patient morbidity, survival, and tumor progression: Clinical article. J. Neurosurg..

[39] Won S.-Y., Dubinski D., Behmanesh B., Bernstock J.D., Seifert V., Konczalla J., Tritt S., Senft C., Gessler F. (2020). Management of hydrocephalus after resection of posterior fossa lesions in pediatric and adult patients—Predictors for development of hydrocephalus. Neurosurg. Rev..

[40] Ebel F., Greuter L., Licci M., Guzman R., Soleman J. (2021). Endoscopic and endoscopically-assisted resection of intraventricular lesions using a neuroendoscopic ultrasonic aspirator. J. Clin. Med..

[41] Wibroe M., Rochat P., Juhler M. (2018). Cerebellar mutism syndrome and other complications after surgery in the posterior fossa in adults: A prospective study. World Neurosurg..

[42] Gleissner U., Sassen R., Schramm J., Elger C.E., Helmstaedter C. (2005). Greater functional recovery after temporal lobe epilepsy surgery in children. Brain.

[43] Boatman D., Freeman J., Vining E., Pulsifer M., Miglioretti D., Minahan R., Carson B., Brandt J., McKhann G. (1999). Language recovery after left hemispherectomy in children with late-onset seizures. Ann. Neurol..

[44] Marsh E.B., Newhart M., Kleinman J.T., Heidler-Gary J., Vining E.P., Freeman J.M., Kossoff E.H., Hillis A.E. (2009). Hemispherectomy sustained before adulthood does not cause persistent hemispatial neglect. Cortex.

[45] Mikellidou K., Arrighi R., Aghakhanyan G., Tinelli F., Frijia F., Crespi S., De Masi F., Montanaro D., Morrone M. (2019). Plasticity of the human visual brain after an early cortical lesion. Neuropsychologia.

[46] Trevisi G., Roujeau T., Duffau H. (2016). Awake surgery for hemispheric low-grade gliomas: Oncological, functional and methodological differences between pediatric and adult populations. Child’s Nerv. Syst..

[47] Johnson D.R., Brown P.D., Galanis E., Hammack J.E. (2012). Pilocytic astrocytoma survival in adults: Analysis of the surveillance, epidemiology, and end results program of the National Cancer Institute. J. Neuro-Oncol..

[48] Laws E.R., Taylor W.F., Clifton M.B., Okazaki H. (1984). Neurosurgical management of low-grade astrocytoma of the cerebral hemispheres. J. Neurosurg..

[49] Fisher B.J., Leighton C.C., Vujovic O., Macdonald D.R., Stitt L. (2001). Results of a policy of surveillance alone after surgical management of pediatric low grade gliomas. Int. J. Radiat. Oncol.*Biol.*Phys..

[50] Saunders D.E., Hayward R.D. (2005). Surveillance imaging strategies following surgery and/or radiotherapy for childhood cerebellar low-grade astrocytoma. J. Neurosurg..

[51] Ater J.L., Zhou T., Holmes E., Mazewski C.M., Booth T.N., Freyer D.R., Lazarus K.H., Packer R.J., Prados M., Sposto R. (2012). Randomized study of two chemotherapy regimens for treatment of low-grade glioma in young children: A report from the Children’s Oncology Group. Clin. Oncol..

[52] Gnekow A.K., Walker D.A., Kandels D., Picton S., Perilongo G., Grill J., Stokland T., Sandstrom P.E., Warmuth-Metz M., Pietsch T. (2017). A European randomised controlled trial of the addition of etoposide to standard vincristine and carboplatin induction as part of an 18-month treatment programme for childhood (≤16 years) low grade glioma—A final report. Eur. J. Cancer.

[53] Stokland T., Liu J.-F., Ironside J.W., Ellison D.W., Taylor R., Robinson K.J., Picton S.V., Walker D.A. (2010). A multivariate analysis of factors determining tumor progression in childhood low-grade glioma: A population-based cohort study (CCLG CNS9702). Neuro-Oncology.

[54] Gogos A.J., Young J.S., Pereira M.P., Morshed R.A., Potts M.B., Hervey-Jumper S.L., Berger M.S. (2020). Surgical management of incidentally discovered low-grade gliomas. J. Neurosurg..

[55] Almekkawi A.K., El Ahmadieh T.Y., Wu E.M., Abunimer A.M., Abi-Aad K.R., Aoun S.G., Plitt A.R., E El Tecle N., Patel T., Stummer W. (2020). The use of 5-aminolevulinic acid in low-grade glioma resection: A systematic review. Oper. Neurosurg..

[56] Zhang C., Boop F.A., Ruge J. (2019). The use of 5-aminolevulinic acid in resection of pediatric brain tumors: A critical review. J. Neurooncol..

[57] Rosca L., Robert-Boire V., Delisle J.-F., Samson Y., Perreault S. (2018). Carboplatin and vincristine neurotoxicity in the treatment of pediatric low-grade gliomas. Pediatr. Blood Cancer.

[58] Merchant T.E., Conklin H.M., Wu S., Lustig R.H., Xiong X. (2009). Late effects of conformal radiation therapy for pediatric patients with low-grade glioma: Prospective evaluation of cognitive, endocrine, and hearing deficits. J. Clin. Oncol..

[59] Packer R.J., Lange B., Ater J., Nicholson H.S., Allen J., Walker R., Prados M., Jakacki R., Reaman G., Needles M.N. (1993). Carboplatin and vincristine for recurrent and newly diagnosed low-grade gliomas of childhood. J. Clin. Oncol..

[60] Nellan A., Wright E., Campbell K., Davies K.D., Donson A.M., Amani V., Judd A., Hemenway M.S., Raybin J., Foreman N.K. (2020). Retrospective analysis of combination carboplatin and vinblastine for pediatric low-grade glioma. J. Neuro-Oncol..

[61] Ater J.L., Xia C., Mazewski C.M., Booth T.N., Freyer D.R., Packer R.J., Sposto R., Vezina G., Pollack I.F. (2016). Nonrandomized comparison of neurofibromatosis type 1 and non-neurofibromatosis type 1 children who received carboplatin and vincristine for progressive low-grade glioma: A report from the Children’s Oncology Group: Low-grade glioma in NF1. Cancer.

[62] Fangusaro J., Onar-Thomas A., Poussaint T.Y., Wu S., Ligon A.H., Lindeman N., Banerjee A., Packer R.J., Kilburn L.B., Goldman S. (2019). Selumetinib in paediatric patients with BRAF-aberrant or neurofibromatosis type 1-associated recurrent, refractory, or progressive low-grade glioma: A multicentre, phase 2 trial. Lancet Oncol..

[63] Selt F., Van Tilburg C.M., Bison B., Sievers P., Harting I., Ecker J., Pajtler K.W., Sahm F., Bahr A., Simon M. (2020). Response to trametinib treatment in progressive pediatric low-grade glioma patients. J. Neurooncol..

[64] Manoharan N., Choi J., Chordas C., Zimmerman M.A., Scully J., Clymer J., Filbin M., Ullrich N.J., Bandopadhayay P., Chi S.N. (2020). Trametinib for the treatment of recurrent/progressive pediatric low-grade glioma. J. Neuro-Oncol..

[65] Greenberger B., Pulsifer M.B., Ebb D.H., MacDonald S.M., Jones R.M., Butler W.E., Huang M.S., Marcus K.J., Oberg J., Tarbell N.J. (2014). Clinical outcomes and late endocrine, neurocognitive, and visual profiles of proton radiation for pediatric low-grade gliomas. Int. J. Radiat. Oncol. Biol. Phys..

[66] Ludmir E.B., Mahajan A., Paulino A.C., Jones J.Y., Ketonen L.M., Su J.M., Grosshans D.R., McAleer M.F., McGovern S.L., A Lassen-Ramshad Y. (2019). Increased risk of pseudoprogression among pediatric low-grade glioma patients treated with proton versus photon radiotherapy. Neuro-Oncology.

[67] Geurts M., van den Bent M.J. (2019). On high-risk, low-grade glioma: What distinguishes high from low? Cancer case conundrums. Cancer.

[68] Brown T.J., A Bota D., Bent M.J.V.D., Brown P.D., Maher E., Aregawi D., Liau L., Buckner J.C., Weller M., Berger M.S. (2019). Management of low-grade glioma: A systematic review and meta-analysis. Neuro-Oncol. Pract..

[69] Chukwueke U.N., Wen P.Y. (2019). Use of the Response Assessment in Neuro-Oncology (RANO) criteria in clinical trials and clinical practice. CNS Oncol..

[70] Van den Bent M.J., Afra D., De Witte O., Hassel M.B., Schraub S., Hoang-Xuan K., Malmström P.-O., Collette L., Piérart M., Mirimanoff R. (2005). Long-term efficacy of early versus delayed radiotherapy for low-grade astrocytoma and oligodendroglioma in adults: The EORTC 22845 randomised trial. Lancet.

[71] Karim A.B., Afra D., Cornu P., Bleehan N., Schraub S., De Witte O., Darcel F., Stenning S., Pierart M., Van Glabbeke M. (2002). Randomized trial on the efficacy of radiotherapy for cerebral low-grade glioma in the adult: European Organization for Research and Treatment of Cancer Study 22845 with the Medical Research Council study BRO4: An interim analysis. Int. J. Radiat. Oncol. Biol. Phys..

[72] Karim A.B., Afra D., Cornu P., Bleehan N., Schraub S., De Witte O., Darcel F., Stenning S., Pierart M., Van Glabbeke M. (2019). Management of pediatric low-grade glioma. Curr. Opin. Pediatr..

[73] Zhang J., Wu G., Miller C.P., Tatevossian R.G., Dalton J.D., Tang B., Orisme W., Punchihewa C., Parker M., Qaddoumi I. (2013). Whole-genome sequencing identifies genetic alterations in pediatric low-grade gliomas. Nat. Genet..

[74] Ryall S., Zapotocky M., Fukuoka K., Nobre L., Stucklin A.S.G., Bennett J., Siddaway R., Li C., Pajovic S., Arnoldo A. (2020). Integrated molecular and clinical analysis of 1000 pediatric low-grade gliomas. Cancer Cell.

[75] Khatua S., Gutmann D.H., Packer R.J. (2018). Neurofibromatosis type 1 and optic pathway glioma: Molecular interplay and therapeutic insights. Pediatr. Blood Cancer.

[76] Peeters S.M., Muftuoglu Y., Na B., Daniels D.J., Wang A.C. (2021). Pediatric gliomas. Neurosurg. Clin. N. Am..

[77] Packer R.J., Pfister S., Bouffet E., Avery R., Bandopadhayay P., Bornhorst M., Bowers D., Ellison D., Fangusaro J., Foreman N. (2016). Pediatric low-grade gliomas: Implications of the biologic era. Neuro-Oncology.

[78] Lassaletta A., Zapotocky M., Mistry M., Ramaswamy V., Honnorat M., Krishnatry R., Stucklin A.S.G., Zhukova N., Arnoldo A., Ryall S. (2017). Therapeutic and prognostic implications of BRAF V600E in pediatric low-grade gliomas. J. Clin. Oncol..

[79] Mackay A., Burford A., Molinari V., Jones D.T.W., Izquierdo E., Brouwer-Visser J., Giangaspero F., Haberler C., Pietsch T., Jacques T.S. (2018). Molecular, pathological, radiological, and immune profiling of non-brainstem pediatric high-grade glioma from the HERBY phase II randomized trial. Cancer Cell.

[80] Hatae R., Hata N., Suzuki S.O., Yoshimoto K., Kuga D., Murata H., Akagi Y., Sangatsuda Y., Iwaki T., Mizoguchi M. (2017). A comprehensive analysis identifies *BRAF* hotspot mutations associated with gliomas with peculiar epithelial morphology: BRAF mutations in epithelioid gliomas. Neuropathology.

[81] Karajannis M.A., Legault G., Fisher M.J., Milla S.S., Cohen K.J., Wisoff J., Harter D.H., Goldberg J.D., Hochman T., Merkelson A. (2014). Phase II study of sorafenib in children with recurrent or progressive low-grade astrocytomas. Neuro-Oncology.

[82] Baumert B.G., Hegi M.E., van den Bent M.J., Deimling A., Gorlia T., Hoang-Xuan K., Brandes A.A., Kantor G., Taphoorn M.J.B., Hassel M.B. (2016). Temozolomide chemotherapy versus radiotherapy in high-risk low-grade glioma (EORTC 22033-26033): A randomised, open-label, phase 3 intergroup study. Lancet Oncol..

[83] Buckner J.C., Shaw E.G., Pugh S.L., Chakravarti A., Gilbert M.R., Barger G.R., Coons S., Ricci P., Bullard D., Brown P.D. (2016). Radiation plus Procarbazine, CCNU, and Vincristine in Low-Grade Glioma. N. Engl. J. Med..

[84] Sievert A.J., Lang S.-S., Boucher K.L., Madsen P.J., Slaunwhite E., Choudhari N., Kellet M., Storm P.B., Resnick A.C. (2013). Paradoxical activation and RAF inhibitor resistance of BRAF protein kinase fusions characterizing pediatric astrocytomas. Proc. Natl. Acad. Sci. USA.

[85] Winograd E., Pencovich N., Yalon M., Soffer D., Beni-Adani L., Constantini S. (2012). Malignant transformation in pediatric spinal intramedullary tumors: Case-based update. Child’s Nerv. Syst..

[86] Ünal E., Koksal Y., Çimen O., Paksoy Y., Tavli L. (2008). Malignant glioblastomatous transformation of a low-grade glioma in a child. Child’s Nerv. Syst..

[87] Van Der Wal E.P., Azzarelli B., Edwards-Brown M. (2003). Malignant transformation of a chiasmatic pilocytic astrocytoma in a patient with diencephalic syndrome. Pediatr. Radiol..

[88] Tom M.C., Park D.Y., Yang K., Leyrer C.M., Wei W., Jia X., Varra V., Yu J.S., Chao S.T., Balagamwala E.H. (2019). Malignant transformation of molecularly classified adult low-grade glioma. Int. J. Radiat. Oncol..

[89] Kortmann R.-D., Jeremic B., Weller M., Lutterbach J., Paulsen F., Bamberg M. (2004). Immediate postoperative radiotherapy or “watch and wait” in the management of adult low-grade glioma?. Strahlenther. Onkol..

[90] Murphy E.S., Leyrer C.M., Parsons M., Suh J.H., Chao S.T., Yu J.S., Kotecha R., Jia X., Peereboom D.M., Prayson R.A. (2018). Risk factors for malignant transformation of low-grade glioma. Int. J. Radiat. Oncol. Biol. Phys..

[91] Hanada T., Rahayu T.U., Yamahata H., Hirano H., Yoshioka T., Arita K. (2016). Rapid malignant transformation of low-grade astrocytoma in a pregnant woman: Malignant transformation of astrocytoma. J. Obstet. Gynaecol. Res..

[92] Schmidt B.T., Hanna A. (2020). Deadly proliferation and transformation of pilocytic astrocytoma in pregnancy. World Neurosurg..

[93] Daras M., Cone C., Peters K.B. (2014). Tumor progression and transformation of low-grade glial tumors associated with pregnancy. J. Neuro-Oncol..

[94] Hirano H., Lopes M.B., Laws ERJr Asakura T., Goto M., Carpenter J.E., Karns L.R., VandenBerg S.R. (1999). Insulin-like growth factor-1 content and pattern of expression correlates with histopathologic grade in diffusely in ltrating astrocytomas. Neuro-Oncology.

[95] Roelvink N.C.A., Kamphorst W., Van Alphen H.A.M., Rao B.R. (1987). Pregnancy-related primary brain and spinal tumors. Arch. Neurol..

[96] Patterson B., Chen Y., Sklar C.A., Neglia J., Yasui Y., Mertens A., Armstrong G.T., Meadows A., Stovall M., Robison L.L. (2014). Growth hormone exposure as a risk factor for the development of subsequent neoplasms of the central nervous system: A report from the childhood cancer survivor study. J. Clin. Endocrinol. Metab..

[97] Leu S., von Felten S., Frank S., Boulay J.-L., Mariani L. (2016). IDH mutation is associated with higher risk of malignant transformation in low-grade glioma. J. Neuro-Oncol..

[98] Jung T.-Y., Jung S., Moon J.-H., Kim I.-Y., Moon K.-S., Jang W.-Y. (2011). Early prognostic factors related to progression and malignant transformation of low-grade gliomas. Clin. Neurol. Neurosurg..

[99] Ogiwara H., Bowman R.M., Tomita T. (2012). Long-term follow-up of pediatric benign cerebellar astrocytomas. Neurosurgery.

[100] Gunny R.S., Hayward R.D., Phipps K.P., Harding B.N., Saunders D.E. (2005). Spontaneous regression of residual low-grade cerebellar pilocytic astrocytomas in children. Pediatr. Radiol..

[101] Palma L., Celli P., Mariottini A. (2004). Long-term follow-up of childhood cerebellar astrocytomas after incomplete resection with particular reference to arrested growth or spontaneous tumour regression. Acta Neurochir..

[102] Tabori U., Vukovic B., Zielenska M., Hawkins C., Braude I., Rutka J., Bouffet E., Squire J., Malkin D. (2006). The role of telomere maintenance in the spontaneous growth arrest of pediatric low-grade gliomas. Neoplasia.

